# Supplementary material for: Preterm disparities between foreign and Swedish born mothers depend on the method used to estimate gestational age. A Swedish population-based register study
Source: PLoS One. 2021 Feb 22;16(2):e0247138. doi: 10.1371/journal.pone.0247138 (PMC7899337; doi:10.1371/journal.pone.0247138)
Supplement: S1 Table — (DOCX) [file pone.0247138.s001.docx]

**S1 Table.** Gestational age outcomes by method of estimation of gestational age by region of origin (corresponding to figures 2 and 3)

| Reference: term births | **Ultrasound** | | | **LMP** | | |  |
| --- | --- | --- | --- | --- | --- | --- | --- |
| (37-41 weeks) | OR | 95% CI | P-value | OR | 95% CI | P values | Consistent |
| **Preterm (<37 weeks)** | |  |  |  |  |  |  |
| Swedish-born (ref) | 1 |  |  | 1 |  |  |  |
| Foreign-born | 0.98 | [0.96,1.01] | 0.242 | 1.10 | [1.07,1.14] | <0.001 | NO |
| Rest of Nordics | 0.94 | [0.87,1.02] | 0.104 | 1.00 | [0.93,1.08] | 0.968 | YES |
| Western Europe & USA | 0.73 | [0.64,0.85] | <0.001 | 0.76 | [0.65,0.88] | <0.001 | YES |
| Eastern EU & Russia | 0.99 | [0.94,1.05] | 0.797 | 1.09 | [1.03,1.15] | 0.003 | NO |
| Middle East | 0.90 | [0.86,0.94] | 0.000 | 1.00 | [0.96,1.05] | 0.922 | NO |
| Africa | 0.87 | [0.80,0.95] | 0.002 | 1.23 | [1.15,1.34] | <0.001 | NO |
| Asia | 1.33 | [1.25,1.41] | <0.001 | 1.48 | [1.40,1.58] | <0.001 | YES |
| Latin America | 1.09 | [0.98,1.21] | 0.117 | 1.12 | [1.00,1.25] | 0.049 | NO |
| **Post-term (>42 weeks)** | |  |  |  |  |  |  |
| Swedish-born (ref) | 1 |  |  | 1 |  |  |  |
| Foreign-born | 0.88 | [0.87,0.89] | <0.001 | 0.89 | [0.88,0.90] | <0.001 | YES |
| Rest of Nordics | 0.96 | [0.93,1.00] | 0.063 | 0.92 | [0.89,0.95] | <0.001 | NO |
| Western Europe & USA | 0.95 | [0.89,1.01] | 0.108 | 0.90 | [0.85,0.95] | <0.001 | NO |
| Eastern EU & Russia | 0.92 | [0.90,0.95] | <0.001 | 0.89 | [0.87,0.92] | <0.001 | YES |
| Middle East | 0.72 | [0.70,0.74] | <0.001 | 0.84 | [0.82,0.86] | <0.001 | YES |
| Africa | 1.93 | [1.87,2.00] | <0.001 | 1.32 | [1.28,1.37] | <0.001 | YES |
| Asia | 0.51 | [0.49,0.54] | <0.001 | 0.72 | [0.69,0.74] | <0.001 | YES |
| Latin America | 0.63 | [0.59,0.67] | <0.001 | 0.81 | [0.77,0.85] | <0.001 | YES |
|  |  |  |  |  |  |  |  |
| **Very preterm (<32 weeks)** | |  |  |  |  |  |  |
| Swedish-born (ref) | 1 |  |  | 1 |  |  |  |
| Foreign-born | 1.13 | [1.07,1.20] | <0.001 | 1.16 | [1.09,1.23] | <0.001 | YES |
| Rest of Nordics | 1.01 | [0.85,1.19] | 0.953 | 1.07 | [0.90,1.27] | 0.375 | YES |
| Western Europe & USA | 0.77 | [0.55,1.07] | 0.124 | 0.73 | [0.51,1.05] | 0.088 | YES |
| Eastern EU & Russia | 1.29 | [1.15,1.44] | 0.000 | 1.34 | [1.20,1.51] | <0.001 | YES |
| Middle East | 0.97 | [0.88,1.08] | 0.601 | 0.99 | [0.88,1.10] | 0.749 | YES |
| Africa | 1.30 | [1.10,1.54] | 0.002 | 1.37 | [1.16,1.62] | <0.001 | YES |
| Asia | 1.31 | [1.14,1.51] | 0.000 | 1.28 | [1.10,1.48] | 0.002 | YES |
| Latin America | 1.34 | [1.08,1.67] | 0.008 | 1.40 | [1.11,1.76] | 0.004 | YES |
| **Moderately preterm (32-36 weeks)** | | |  |  |  |  |  |
| Swedish-born (ref) | 1 |  |  | 1 |  |  |  |
| Foreign-born | 0.95 | [0.92,0.98] | 0.002 | 1.09 | [1.06,1.13] | <0.001 | NO |
| Rest of Nordics | 0.93 | [0.86,1.00] | 0.065 | 0.99 | [0.91,1.07] | <0.001 | YES |
| Western Europe & USA | 0.73 | [0.62,0.85] | 0.000 | 0.76 | [0.65,0.89] | <0.001 | YES |
| Eastern EU & Russia | 0.93 | [0.88,0.99] | 0.022 | 1.03 | [0.97,1.10] | <0.001 | NO |
| Middle East | 0.88 | [0.84,0.93] | 0.000 | 1.00 | [0.96,1.06] | <0.001 | NO |
| Africa | 0.78 | [0.70,0.86] | 0.000 | 1.20 | [1.11,1.31] | <0.001 | NO |
| Asia | 1.33 | [1.25,1.42] | 0.000 | 1.52 | [1.43,1.63] | <0.001 | YES |
| Latin America | 1.03 | [0.92,1.16] | 0.564 | 1.06 | [0.94,1.19] | <0.001 | YES |
| N | 1,317,265 |  |  | 1,317,265 |  |  |  |

OR= Odd Ratios; CI= Confidence Intervals
