# Supplementary material for: Preterm disparities between foreign and Swedish born mothers depend on the method used to estimate gestational age. A Swedish population-based register study
Source: PLoS One. 2021 Feb 22;16(2):e0247138. doi: 10.1371/journal.pone.0247138 (PMC7899337; doi:10.1371/journal.pone.0247138)
Supplement: S2 Table — (DOCX) [file pone.0247138.s002.docx]

**S2 Table.** Preterm according to LMP and ultrasound estimates by mother’s country of birth.

| **Reference: term births** |  | **Ultrasound** |  |  | **LMP** |  |  |  |
| --- | --- | --- | --- | --- | --- | --- | --- | --- |
| **(37-41 weeks)** |  | **OR** | **95% CI** | **P values** | **OR** | **95% CI** | **P values** | **Consistent** |
|  | Swedish-born (ref) | 1 |  |  | 1 |  |  |  |
| Rest of Nordics | Denmark | 0.89 | [0.73,1.08] | 0.232 | 1.03 | [0.85,1.24] | 0.776 | YES |
|  | Finland | 0.98 | [0.89,1.07] | 0.568 | 1.05 | [0.96,1.15] | 0.305 | YES |
|  | Norway | 0.89 | [0.76,1.04] | 0.138 | 0.84 | [0.70,1.00] | 0.047 | YES |
| Western Europe  & USA | Germany | 0.71 | [0.59,0.87] | 0.001 | 0.71 | [0.58,0.87] | 0.001 | YES |
|  | UK | 0.86 | [0.63,1.16] | 0.318 | 0.96 | [0.71,1.29] | 0.795 | YES |
|  | US | 0.67 | [0.50,0.91] | 0.009 | 0.68 | [0.50,0.92] | 0.014 | YES |
| Eastern Europé  & Russia | F.Yugoslavia | 0.96 | [0.90,1.03] | 0.285 | 1.09 | [1.02,1.17] | 0.012 | NO |
|  | Poland | 1.02 | [0.91,1.14] | 0.668 | 1.09 | [0.97,1.23] | 0.127 | YES |
|  | Romania | 1.19 | [0.97,1.46] | 0.084 | 1.14 | [0.93,1.41] | 0.215 | YES |
|  | Hungry | 0.98 | [0.72,1.33] | 0.889 | 1.03 | [0.75,1.41] | 0.870 | YES |
|  | Russia | 0.99 | [0.81,1.22] | 0.987 | 0.99 | [0.80,1.24] | 0.965 | YES |
| Middle East | Turkey | 0.91 | [0.82,1.02] | 0.121 | 0.98 | [0.88,1.10] | 0.800 | YES |
|  | Lebanon | 0.80 | [0.70,0.90] | <0.001 | 0.91 | [0.81,1.04] | 0.157 | YES |
|  | Syria | 0.76 | [0.66,0.88] | <0.001 | 0.84 | [0.72,0.97] | 0.019 | YES |
|  | Iraq | 0.89 | [0.83,0.96] | 0.002 | 1.01 | [0.94,1.09] | 0.722 | YES |
|  | Iran | 1.09 | [0.98,1.22] | 0.110 | 1.12 | [1.00,1.26] | 0.045 | NO |
|  | Afghanistan | 0.87 | [0.69,1.09] | 0.241 | 1.18 | [0.96,1.46] | 0.116 | YES |
|  | Pakistan | 1.03 | [0.80,1.34] | 0.771 | 1.26 | [0.99,1.61] | 0.052 | NO |
| Africa | Eritrea | 0.89 | [0.70,1.14] | 0.391 | 1.32 | [1.07,1.64] | 0.009 | NO |
|  | Ethiopia | 0.88 | [0.72,1.08] | 0.214 | 1.27 | [1.06,1.51] | 0.008 | NO |
|  | Somalia | 0.93 | [0.82,1.06] | 0.271 | 1.36 | [1.23,1.51] | <0.001 | NO |
|  | Morocco | 0.73 | [0.57,0.94] | 0.014 | 0.81 | [0.63,1.04] | 0.105 | NO |
|  | Tunisia | 0.57 | [0.37,0.86] | 0.008 | 0.78 | [0.53,1.14] | 0.196 | NO |
| Asia | SriLanka | 1.64 | [1.34,2.00] | <0.001 | 1.49 | [1.20,1.86] | <0.001 | YES |
|  | Philippines | 1.52 | [1.29,1.78] | <0.001 | 1.75 | [1.49,2.05] | <0.001 | YES |
|  | India | 1.87 | [1.62,2.16] | <0.001 | 1.74 | [1.49,2.03] | <0.001 | YES |
|  | Thailand | 1.21 | [1.07,1.36] | 0.002 | 1.50 | [1.34,1.69] | <0.001 | YES |
|  | Vietnam | 1.1 | [0.92,1.31] | 0.301 | 1.65 | [1.41,1.92] | <0.001 | NO |
|  | China | 0.89 | [0.72,1.10] | 0.277 | 0.90 | [0.73,1.13] | 0.375 | YES |
|  | Korea | 1.17 | [0.97,1.41] | 0.093 | 1.22 | [1.01,1.48] | 0.036 | NO |
|  | Bangladesh | 1.76 | [1.40,2.22] | <0.001 | 1.50 | [1.15,1.94] | 0.002 | YES |
| Latin America | Chile | 1.10 | [0.96,1.26] | 0.169 | 1.14 | [0.99,1.32] | 0.074 | YES |
|  | Brasil | 0.98 | [0.73,1.32] | 0.891 | 0.92 | [0.67,1.27] | 0.629 | YES |
|  | Peru | 0.83 | [0.60,1.16] | 0.282 | 0.88 | [0.64,1.21] | 0.433 | YES |
|  | Colombia | 1.35 | [1.05,1.73] | 0.017 | 1.41 | [1.10,1.82] | 0.007 | YES |
|  | N | 1,317,265 |  |  | 1,317,265 |  |  |  |
