# Supplementary material for: Preterm disparities between foreign and Swedish born mothers depend on the method used to estimate gestational age. A Swedish population-based register study
Source: PLoS One. 2021 Feb 22;16(2):e0247138. doi: 10.1371/journal.pone.0247138 (PMC7899337; doi:10.1371/journal.pone.0247138)
Supplement: S3 Table — (DOCX) [file pone.0247138.s003.docx]

**S3 Table.** Post-term according to LMP and ultrasound estimates by mother’s country of birth.

| Reference: term births (37-41 weeks) |  | Ultrasound | |  | LMP |  |  | Consistent |
| --- | --- | --- | --- | --- | --- | --- | --- | --- |
|  |  | OR | 95% CI | P values | OR | 95% CI | P values |  |
|  | Swedish-born (ref) | 1 |  |  | 1 |  |  |  |
| Nordic | Denmark | 1.03 | [0.94,1.12] | 0.541 | 1 | [0.94,1.08] | 0.897 | YES |
|  | Finland | 0.92 | [0.87,0.96] | 0.001 | 0.87 | [0.83,0.90] | 0.000 | YES |
|  | Norway | 1.05 | [0.97,1.14] | 0.222 | 1 | [0.93,1.06] | 0.929 | YES |
| Western Europe & USA | Germany | 0.90 | [0.82,0.98] | 0.022 | 0.87 | [0.80,0.94] | <0.001 | YES |
|  | UK | 1.04 | [0.91,1.19] | 0.530 | 0.99 | [0.88,1.10] | 0.824 | YES |
|  | US | 0.97 | [0.85,1.11] | 0.657 | 0.89 | [0.80,0.99] | 0.038 | YES |
| Eastern Europe & Russia | F.Yugoslavia | 0.93 | [0.90,0.97] | <0.001 | 0.91 | [0.88,0.94] | <0.001 | NO |
|  | Poland | 0.94 | [0.88,1.00] | 0.035 | 0.90 | [0.85,0.94] | <0.001 | YES |
|  | Romania | 0.79 | [0.70,0.90] | <0.001 | 0.76 | [0.69,0.84] | <0.001 | YES |
|  | Hungry | 0.73 | [0.61,0.88] | 0.001 | 0.84 | [0.73,0.96] | 0.013 | YES |
|  | Russia | 0.98 | [0.88,1.10] | 0.740 | 0.92 | [0.83,1.00] | 0.058 | YES |
| Middle East | Turkey | 0.72 | [0.68,0.77] | <0.001 | 0.84 | [0.80,0.88] | <0.001 | YES |
|  | Lebanon | 0.78 | [0.73,0.84] | <0.001 | 0.89 | [0.84,0.94] | <0.001 | YES |
|  | Syria | 0.67 | [0.62,0.73] | <0.001 | 0.78 | [0.73,0.83] | <0.001 | YES |
|  | Iraq | 0.72 | [0.69,0.75] | <0.001 | 0.89 | [0.86,0.92] | <0.001 | YES |
|  | Iran | 0.70 | [0.65,0.75] | <0.001 | 0.73 | [0.69,0.77] | <0.001 | NO |
|  | Afghanistan | 0.70 | [0.62,0.80] | <0.001 | 0.90 | [0.81,1.00] | 0.043 | YES |
|  | Pakistan | 0.58 | [0.49,0.69] | <0.001 | 0.77 | [0.68,0.87] | <0.001 | NO |
| Africa | Eritrea | 1.88 | [1.70,2.09] | <0.001 | 1.20 | [1.09,1.31] | <0.001 | YES |
|  | Ethiopia | 1.86 | [1.71,2.01] | <0.001 | 1.28 | [1.19,1.38] | <0.001 | YES |
|  | Somalia | 2.24 | [2.14,2.35] | <0.001 | 1.49 | [1.42,1.55] | <0.001 | YES |
|  | Morocco | 1.29 | [1.16,1.44] | <0.001 | 1.03 | [0.94,1.13] | 0.509 | NO |
|  | Tunisia | 1.07 | [0.90,1.29] | 0.447 | 0.98 | [0.85,1.14] | 0.816 | YES |
| Asia | SriLanka | 0.56 | [0.47,0.66] | <0.001 | 0.68 | [0.60,0.77] | <0.001 | YES |
|  | Philippines | 0.44 | [0.39,0.51] | <0.001 | 0.65 | [0.59,0.71] | <0.001 | YES |
|  | India | 0.51 | [0.45,0.59] | <0.001 | 0.70 | [0.64,0.77] | <0.001 | YES |
|  | Thailand | 0.50 | [0.46,0.55] | <0.001 | 0.72 | [0.67,0.76] | <0.001 | YES |
|  | Vietnam | 0.29 | [0.25,0.34] | <0.001 | 0.67 | [0.61,0.73] | <0.001 | YES |
|  | China | 0.65 | [0.57,0.74] | <0.001 | 0.74 | [0.68,0.82] | <0.001 | YES |
|  | Korea | 0.71 | [0.63,0.79] | <0.001 | 0.82 | [0.76,0.90] | <0.001 | YES |
|  | Bangladesh | 0.54 | [0.44,0.66] | <0.001 | 0.81 | [0.71,0.93] | 0.003 | YES |
| Latin America | Chile | 0.53 | [0.49,0.59] | <0.001 | 0.84 | [0.79,0.89] | <0.001 | YES |
|  | Brasil | 0.93 | [0.80,1.10] | 0.404 | 0.83 | [0.73,0.95] | 0.008 | YES |
|  | Peru | 0.80 | [0.67,0.95] | 0.009 | 0.71 | [0.62,0.82] | <0.001 | YES |
|  | Colombia | 0.59 | [0.49,0.71] | <0.001 | 0.76 | [0.67,0.87] | <0.001 | YES |
|  | N | 1,317,265 |  |  | 1,317,265 |  |  |  |
