# Supplementary material for: Preterm disparities between foreign and Swedish born mothers depend on the method used to estimate gestational age. A Swedish population-based register study
Source: PLoS One. 2021 Feb 22;16(2):e0247138. doi: 10.1371/journal.pone.0247138 (PMC7899337; doi:10.1371/journal.pone.0247138)
Supplement: S4 Table — Gestational age outcomes according to LMP and ultrasound estimates in a subsample of 718,191 of uncomplicated pregnancies. (DOCX) [file pone.0247138.s004.docx]

**S4 Table**. Sensitivity analyses. Gestational age outcomes according to LMP and ultrasound estimates in a subsample of 718,191 of uncomplicated pregnancies.

| **Reference: term births** | **Ultrasound** | |  | **LMP** |  |  |  |
| --- | --- | --- | --- | --- | --- | --- | --- |
| **(37-41 weeks)** | **OR** | **95% CI** | **P-value** | **OR** | **95% CI** | **P-value** | **Consistent** |
| Swedish-born (ref) | 1 |  |  | 1 |  |  |  |
| **Preterm (<37 weeks)** |  |  |  |  |  |  |  |
| Foreign-born | 0.90 | [0.86,0.94] | <0.001 | 1.07 | [1.02,1.12] | 0.005 | NO |
| **Post-term (>42 weeks)** |  |  |  |  |  |  |  |
| Foreign-born | 0.90 | [0.88,0.91] | <0.001 | 0.91 | [0.89,0.92] | <0.001 | YES |
|  |  |  |  |  |  |  |  |
| **Very preterm**  **(<32 weeks)** |  |  |  |  |  |  |  |
| Foreign-born | 1.15 | [1.01,1.32] | 0.038 | 1.17 | [1.02,1.35] | 0.028 | YES |
| **Moderately preterm (32-36 weeks)** |  |  |  |  |  |  |  |
| Foreign-born | 0.87 | [0.83,0.92] | <0.001 | 1.06 | [1.01,1.12] | 0.015 | NO |
| N | 718,191 |  |  | 718,191 |  |  |  |

OR= Odd Ratios; CI= Confidence Intervals.

Note: models adjusted for year of birth.
