# Supplementary material for: Preterm disparities between foreign and Swedish born mothers depend on the method used to estimate gestational age. A Swedish population-based register study
Source: PLoS One. 2021 Feb 22;16(2):e0247138. doi: 10.1371/journal.pone.0247138 (PMC7899337; doi:10.1371/journal.pone.0247138)
Supplement: S5 Table — Gestational age outcomes according to LMP and ultrasound estimates including 723,253 missing in different covariates. (DOCX) [file pone.0247138.s005.docx]

**S5 Table.** Sensitivity analyses. Gestational age outcomes according to LMP and ultrasound estimates including 723,253 missing in different covariates.

| **Reference: term births** | **Ultrasound** | |  | **LMP** |  |  | **Consistent** |
| --- | --- | --- | --- | --- | --- | --- | --- |
| **(37-41 weeks)** | **OR** | **95% CI** | **P-value** | **OR** | **95% CI** | **P-value** |  |
| Swedish-born (ref) | **1** |  |  | **1** |  |  |  |
| **Preterm (<37 weeks)** |  |  |  |  |  |  |  |
| Foreign-born | 0.99 | [0.97,1.01] | 0.364 | 1.10 | [1.08,1.12] | <0.001 | NO |
| **Post-term (>42 weeks)** |  |  |  |  |  |  |  |
| Foreign-born | 0.88 | [0.87,0.90] | <0.001 | 0.89 | [0.89,0.91] | <0.001 | YES |
|  |  |  |  |  |  |  |  |
|  |  |  |  |  |  |  |  |
| **Very preterm**  **(<32 weeks)** |  |  |  |  |  |  |  |
| Foreign-born | 1.11 | [1.05,1.17] | <0.001 | 1.13 | [1.07,1.19] | <0.001 | YES |
| **Moderately preterm (32-36 weeks)** |  |  |  |  |  |  |  |
| Foreign-born | 0.96 | [0.94,0.99] | 0.010 | 1.09 | [1.07,1.12] | <0.001 | NO |
| N | 1,574,090 | |  | 1,574,090 | |  |  |

OR= Odd Ratios; CI= Confidence Intervals.

Note: models adjusted for year of birth.
