# Supplementary material for: Preterm disparities between foreign and Swedish born mothers depend on the method used to estimate gestational age. A Swedish population-based register study
Source: PLoS One. 2021 Feb 22;16(2):e0247138. doi: 10.1371/journal.pone.0247138 (PMC7899337; doi:10.1371/journal.pone.0247138)
Supplement: S6 Table — (DOCX) [file pone.0247138.s006.docx]

**S6 Table.** **Gestational age outcomes by method of estimation of gestational age by region of origin adjusted for maternal variables (corresponding to figures 4)**

| **Reference: term births** | **Ultrasound** | | | **LMP** | | |  |
| --- | --- | --- | --- | --- | --- | --- | --- |
| **(37-41 weeks)** | **OR** | **95% CI** | **P-value** | **OR** | **95% CI** | **P values** | **Consistent** |
| **Preterm (<37 weeks)** |  |  |  |  |  |  |  |
| Swedish-born (ref) | 1 |  |  | 1 |  |  |  |
| Foreign-born | 0.99 | [0.96,1.03] | 0.715 | 1.04 | [1.01,1.08] | 0.017 | NO |
| **Post-term (>42 weeks)** |  |  |  |  |  |  |  |
| Swedish-born (ref) |  |  |  |  |  |  |  |
| Foreign-born | 0.96 | [0.94,0.97] | <0.001 | 0.90 | [0.89,0.92] | <0.001 | YES |
| **Very preterm (<32 weeks)** |  |  |  |  |  |  |  |
| Swedish-born (ref) |  |  |  |  |  |  |  |
| Foreign-born | 1.03 | [0.95,1.11] | 0.505 | 1.03 | [0.95,1.12] | 0.446 | YES |
| **Moderately preterm (32-36 weeks)** |  |  |  |  |  |  |  |
| Swedish-born (ref) |  |  |  |  |  |  |  |
| Foreign-born | 0.99 | [0.95,1.03] | 0.468 | 1.05 | [1.01,1.09] | 0.020 | NO |
| N | 1,317,265 |  |  | 1,317,265 |  |  |  |

OR= Odd Ratios; CI= Confidence Intervals.

Note: models adjusted for year of birth, newborn’s sex, parity, maternal heigh, maternal BMI, maternal age, mother’s educational attainment, household disposable income, marital status, father’s origin, smoking during pregnancy.
